# Condition-Invariant Robot Localization Using Global Sequence Alignment of Deep Features

**DOI:** 10.3390/s21124103

**Published:** 2021-06-15

**Authors:** Junghyun Oh, Changwan Han, Seunghwan Lee

**Affiliations:** 1 Department of Robotics, Kwangwoon University, Seoul 01897, Korea; jhyunoh@kw.ac.kr (J.O.); hcw511@naver.com (C.H.); 2 Department of Electronic Engineering, Kumoh National Institute of Technology, Gumi, Gyeongbuk 39177, Korea

**Keywords:** robotics, localization, sequence alignment, place recognition, deep learning

## Abstract

Localization is one of the essential process in robotics, as it plays an important role in autonomous navigation, simultaneous localization, and mapping for mobile robots. As robots perform large-scale and long-term operations, identifying the same locations in a changing environment has become an important problem. In this paper, we describe a robust visual localization system under severe appearance changes. First, a robust feature extraction method based on a deep variational autoencoder is described to calculate the similarity between images. Then, a global sequence alignment is proposed to find the actual trajectory of the robot. To align sequences, local fragments are detected from the similarity matrix and connected using a rectangle chaining algorithm considering the robot’s motion constraint. Since the chained fragments provide reliable clues to find the global path, false matches on featureless structures or partial failures during the alignment could be recovered and perform accurate robot localization in changing environments. The presented experimental results demonstrated the benefits of the proposed method, which outperformed existing algorithms in long-term conditions.

## 1. Introduction

Visual place recognition that identifies the same locations between a query and database image sequence is a prerequisite for various robotic applications such as navigation and simultaneous localization and mapping (SLAM) [1,2,3,4,5]. Recent studies have focused on place recognition in changing environments, as autonomous robots should perform large-scale and long-term operations. One of the major challenges for the vision-based place recognition is *appearance changes* caused by variations in weather conditions, time of day, or seasons.

To overcome the appearance change problem, visual place recognition systems can usually be divided into two stages [2,6]. The first stage is a visual front-end that extracts features from the image data to compute similarities between observations, and the second stage is a stochastic back-end that determines the most likely path sequence of a robot by comparing the incoming front-end data. This paper presents a robust feature extraction method using a deep architecture in first part, and a novel sequence alignment algorithm to perform precise localization in second part.

Global descriptors that describe the whole image have been used since they have shown more robust performances than local features in changing conditions [7,8]. Recently, learning-based approaches were widely applied to place recognition under substantial appearance changes, and various deep learning frameworks have been employed to extract features from images [9,10,11,12,13]. In this paper, we present a feature extraction method using a *variational autoencoder* (VAE) [14], one of the powerful deep generative models for feature extraction. Since this model learns to compress the input image in a probabilistic way, the extracted codes contain useful information and can be used to build a similarity matrix that represents similarity between images.

After generating the similarity matrix, the most likely path sequence of the robot should be estimated from the matrix to perform precise localization. These sequence-based approaches achieved significant improvements in place recognition by attempting to match sequences rather than single images [15,16]. However, they have a common limitation in that if an incorrect local match occurs, the correct global alignment cannot be recovered. To overcome the problem, we propose *glocal sequence alignment*, a combination of the global and local alignment methods, which arranges the sequences of features to perform precise localization.

**Figure 1:**
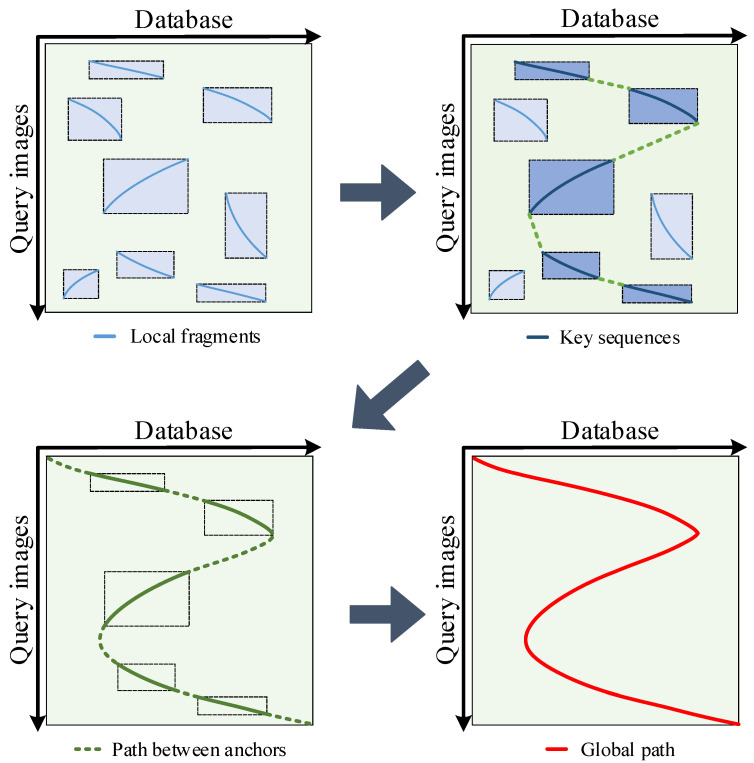
The procedure of the proposed approach. First, local fragments are detected from the similarity matrix using the local alignment method (Section 3.2). Second, the subset of the local alignments is found by the rectangle chaining algorithm that maximizes the total similarity under the motion constraint of the mobile robot (Section 3.3). Then, the path between anchors are calculated using the local alignment method. Finally, the most likely path of the robot is determined using the global aligner (Section 3.4).

The overall procedure of the proposed algorithm is shown in Figure 1. Given a similarity matrix by comparing the deep learning features, local fragments that have local maximum scores are detected from the matrix. Then, reliable ones are found by the rectangle chaining algorithm under the motion constraint of the mobile robot. Finally, the most likely path of the robot is determined using the global aligner. As the chained fragments provide reliable clues to find the global path, false matching on featureless structures or partial failures during the alignment could be recovered.

The present research paper is organized as follows. Section 2 explains related work on place recognition in changing environments. The proposed methodology is explained in Section 3. The feature extraction method from the VAE and the proposed glocal sequence algorithm is discussed in this section. Section 4 presents the validation of the proposed method through publicly available datasets with other algorithms. Finally, Section 5 concludes the paper.

## 2. Related Work

The bag-of-words model from local features such as SIFT [17], SURF [18], and BRIEF [19] has been widely applied to visual place recognition tasks [20,21,22], as they are robust to viewpoint changes. Each image is quantized into a finite number of visual words and can be represented by histograms that can be compared efficiently using Hamming distance or histogram comparison methods. These methods have the advantage of being able to quickly and efficiently recognize a place in a static environment, but have a fatal weakness that false positives can occur in a changing environment.

To overcome the false positive problems, place recognition systems based on global descriptors have been proposed. Unlike the local features, the global descriptors use predefined keypoints and extract information from the whole image. This characteristic makes it possible to distinguish places even if some of image features are similar, and global descriptor based place recognition system has the advantage of being more robust against false positives than local features. Badino et al. [8] proposed whole-image descriptors based on SURF (WI-SURF) to perform place recognition. Similarly, BRIEF-Gist [7] used BRIEF to extract features from the whole image. GIST is one of the popular global descriptors [23] and is widely used in place recognition [24,25,26]. GIST is based on Gabor filters at different orientations and frequencies to extract various information from the image. These results are averaged to generate a compact meaningful vector. GIST is applied in [24] to capture the basic structure of different types of scenes in a compact way from the portions of panoramic images.

Since deep learning, especially convolutional neural network, showed high performance in image classification and recognition, global image descriptors using CNN have been proposed for visual place recognition [10,27,28]. Naseer et al. extracted a sequence of image descriptors using CNNs to compute the similarity matrix and compute matching hypotheses to find loop closures [27]. Sünderhauf et al. first extracted landmark proposals and utilized CNNs features as landmark descriptors [28]. Performances of the deep learned features are evaluated in [10], and the output features of each layer are compared to find the adequate layer for place recognition. Since those approaches used pretrained CNNs such as AlexNet [29] or LeNet [30] for feature extraction, they showed improved performances in changing environments without requiring any training procedure.

To more actively cope with the changing environment, there have been learning-based methods that directly learn a relationship between environments rather than using the pretrained neural network [9,31]. Neubert et al. proposed the concept of appearance change prediction between two different seasons using vocabularies of superpixels [31]. Despite its novelty, the proposed method relied on handcrafted features and segmentation parameters. Lowry et al. also proposed a supervised and an unsupervised learning method for place recognition in changing environments [9]. The supervised learning method depended on linear regression, which finds a linear transform between the two image sequences to predict environmental changes, and unsupervised learning method tried to remove appearance changes based on principal component analysis (PCA). NetVLAD [32] achieved state-of-the-art performance in place recognition by using the CNN and vector of locally aggregated descriptors (VLAD) but takes a large amount of time to perform model training. Oh and Lee proposed a simple convolutional autoencoder (CAE) to recognize places under extreme perceptual changes [33]. Similar to this idea, this paper proposes a feature extraction method based on a VAE, which is a likelihood-based generative model. Since this structure learns mapping from input data to low-dimensional latent vectors in a probabilistic way, features from VAE contain a lot of information of the images even in the low-dimensional vector.

After extracting condition-robust features, the most likely path should be determined by finding correspondences between them. Sequence-based approaches are widely used techniques exploiting the temporal information of image sequences [1]. Milford demonstrated that matching a sequence of images rather than a single image achieved improved performances under extreme perceptual changes [16]. However, a critical limitation of the system is a constant velocity assumption which is often violated in practice. To consider speed variations, Naseer et al. proposed minimum cost network flow in a data association graph [34]. Similarly, Viterbi algorithm [35] and dynamic programming (DP) approach [33] were proposed to determine the most likely path through the environment. Recently, DeepSeqSLAM, a trainable architecture combining CNN and a recurrent neural network (RNN), was proposed to learn visual and positional information from an image sequence [36], and SeqNet proposed hierarchical recognition system using learned short sequential descriptors [37]. However, these methods still have two limitations in common: (1) they are likely to find false matches on featureless structures such as tunnels, corridors, and walls and (2) there is no chance to recover the global alignment once incorrect local matches have occurred.

To overcome the problems, a novel *glocal* sequence alignment method for place recognition is presented, inspired by gene sequence matching of bioinformatics [38]. It is a combination of the global and local sequence alignment that can overcome partial failures. As the proposed method first detects reliable parts and calculates the global path by chaining them, it is able to not only find the accurate matches on featureless environments but also recover the global path even if incorrect local matching occurred.

## 3. Proposed Approach

### 3.1. Similarity Matrix Generation from Deep Learning Features

VAE is a specific type of a neural network that can compress data into the latent vectors in an unsupervised way. Using the latent vector as an image descriptor, the similarity between images can be calculated. The VAE consists of a standard autoencoder component that embeds the input data x into latent codes z by minimizing reconstruction error, and a Bayesian regularization over the latent space, which enforces the posterior of the hidden code vector, matches a prior distribution.

**Figure 2:**
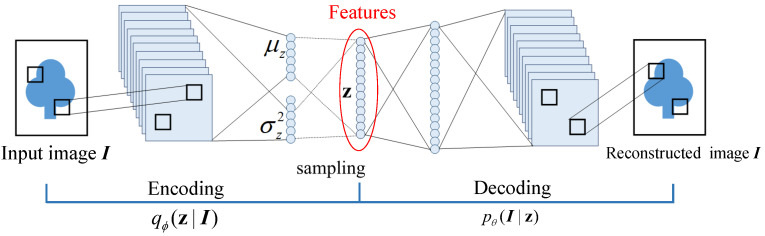
The structure of the VAE that is composed of the encoder and the decoder part.

Let us consider the feature z which compresses the information of the image I. Then the feature z is assumed to generated from prior distribution $p_{\theta }\left(z\right)$, and an image I is generated from some conditional distribution $p_{\theta }\left(I|z\right)$. A recognition model $q_{\phi }\left(z|I\right)$ which is an approximation to the intractable true posterior $p_{\theta }\left(I|z\right)$ is introduced to efficiently approximate posterior inference of the latent variable z given an observed value I for a choice of parameters θ. The recognition model $q_{\phi }\left(z|I\right)$ is also referred as a probabilistic *encoder*, since given a datapoint I, it produces a distribution over the possible values of the code z from which the datapoint I could have been generated. In a similar vein, $p_{\theta }\left(I|z\right)$ is a probabilistic *decoder*, since given a code z, it produces a distribution over the possible corresponding values of I. The structure of the VAE is shown in Figure 2.

The loss function L(θ,ϕ;I) used to train the VAE is the sum of the reconstruction error and the KL-divergence [14] as in the following:

(1) $$L\left(\theta ,\phi ;I\right)=\;E_{q_{\phi }\left(z|I\right)}\left[\log p_{\theta }\left(I|z\right)\right]-D_{KL}(q_{\phi }\left(z|I\right)\left|\right|p_{\theta }\left(z\right)).$$

Training is performed to minimize the loss function L(θ,ϕ;I), and the parameters of the neural network θ and ϕ can be found from solving the optimization problem.

After finishing the training procedure, an input image I is transformed to reconstruct the output image, and outputs of the intermediate layers z can be used as the compressed representation of the image. As these features contain entire information of the images, they are useful for calculating image similarities. If there are two features $z_{i}$ and $z_{j}$ from different images, the similarity score $S_{ij}$ between them is calculated using the cosine similarity.

(2) $$S_{ij}=\frac{z_{i}\cdot z_{j}}{∥z_{i}∥∥z_{j}∥}$$

Suppose there are *M* query and *N* database images from different environments. Then, a similarity matrix $S\in R^{M\times N}$ can be constructed from these similarity scores where each element $S_{ij}$ is the similarity between two images *i* and *j*.

### 3.2. Finding Local Fragments from Similarity Matrix

After generating the similarity matrix, local fragments that are candidates for the global path should be detected. To find the fragments, multiple seeds whose similarity scores are above the threshold τ are found from the *S*. A local sequence alignment method such as Smith–Waterman algorithm [39] is then performed from these points to find local fragments.

The local sequence alignment works through the following procedures. First, the score matrix H is constructed recursively from the similarity matrix S. H is initialized with zeros and filled recursively based on the neighbor’s similarity score and the gap penalty. In this paper, H is constructed using the modified version of scoring method in [33]. The main difference is that negative scores are not allocated in our model to enable local alignment as the following:

(3) $$H_{i,j}=logS_{i,j}+\max_{k\in W(j)}(H_{i-1,k}+\log\delta \left(i,j,k\right),0)$$

where $W\left(j\right)=\left[j-V_{max},j+V_{max}\right]$ is a constrained candidate set, and δ(i,j,k) is the likelihood of transitioning from state *k* to state *j* when the robot’s maximum velocity is $V_{max}$.

Then, the local path is found by tracing back. Starting from the maximum value of the H to the end of zero, the best local alignment is found by tracking the source of each score recursively. Since we have multiple seeds, multiple local sequences can be detected from the S.

Let the *n*-th local fragment has the starting point $\left(x_{i}\left(n\right),y_{i}\left(n\right)\right)$ and the ending point $\left(x_{f}\left(n\right),y_{f}\left(n\right)\right)$. Then, it can be modeled as a rectangle R(n) with these points as diagonal components and define a weight score w(n) which is a length between these points. A rectangle chain of maximum score should be found given a set of these weighted rectangles.

### 3.3. Rectangle Chaining Algorithm

Finding a rectangle chain of maximum score is equivalent to find the indices of rectangles $L=\{p_{1},\cdots ,p_{L}\}$, maximizing the global sequence score as the following equation:

(4) $$L=\underset{p_{1},\cdots ,p_{L}}{\arg\max}\left(\sum_{l=1}^{L}w\left(p_{l}\right)-\sum_{m=1}^{L-1}\delta \left(p_{m},p_{m+1}\right)\right)$$

where δ(u,v) is the gap penalty for connecting R(u) to R(v) in the chain.

To calculate a gap penalty, a new method is proposed considering the motion constraints of the robot. The linear motion model of the robot satisfies $x_{n}=Fx_{n-1}+w$, where x is the state vector, F is the state transition matrix, and w is the process noise drawn from a multivariate Gaussian w∼N(0,Q). Let the state vector contains the position and velocity information as $x_{n}={\left[p_{n},v_{n}\right]}^{T}$, then the equation can be rewritten as follows:

(5) $$\left[\begin{array}{c} p_{n}\\ v_{n} \end{array}\right]=\;\left[\begin{array}{cc} I & \;tI\\ 0 & \;I \end{array}\right]\left[\begin{array}{c} p_{n-1}\\ v_{n-1} \end{array}\right]+w$$

where *t* is the sampling time. Then, the predicted state after *n* steps follows $x_{n}∼N\left(\mu_{n},\Sigma_{n}\right)$ derived as follows:

(6) $$\left\{\begin{array}{cc} \mu_{n} & =F^{n}\mu_{0}\\ \Sigma_{n} & =F^{n}\Sigma_{0}{\left(F^{⊤}\right)}^{n}+\sum_{k=0}^{n-1}F^{k}Q{\left(F^{⊤}\right)}^{k} \end{array}\right.$$

To calculate the δ(u,v), the step size *n* is set to $|y_{f}\left(u\right)-y_{i}\left(v\right)|$, since the initial state of the robot is at the ending point of R(u) and the final state is at the starting point of R(v). Then, the gap penalty finally becomes the following equation:

(7) $$\delta \left(u,v\right)=\;C|\Sigma_{\eta }|\exp{\left(-\frac{1}{2}({\left(x_{v}-\mu_{\eta }\right)}^{⊤}\Sigma_{\eta }^{-1}\left(x_{v}-\mu_{\eta }\right))\right)}^{-1}$$

where $\eta =|y_{f}\left(u\right)-y_{i}\left(v\right)|$, $x_{v}$ is the state at R(v), and *C* is the weight factor. The value of the gap penalty δ(u,v) becomes larger as the distance between R(u) and R(v) increases. Therefore, the gap should be minimized as possible to maximize the score when chaining the rectangles. The details are described in Appendix A.

The rectangle chaining problem under the gap penalty was first introduced in gene sequence matching [38] to chain the ordered local gene sequences. The idea was based on the *sparse DP* which finds the maximum weight chain by comparing the rectangles in the list. The rectangle chaining algorithm is modified considering the motion constraint of the robot.

A new rectangle is only searched through the query sequence, and the score is evaluated based on the combination of weight scores and the gap penalty. Detail procedure is described in Algorithm 1.
**Algorithm 1** Proposed rectangle chaining algorithm.**Input**A set of rectangles R(1),⋯,R(N)**Output**The optimal chaining path $P^{*}$1:**for**t=1 to *T*2:**if** $t=y_{i}\left(k\right)$ of rectangle R(k)3:j← rectangle in L, with largest $y_{f}\left(j\right)<y_{i}\left(k\right)$4:V(k)←w(k)+V(j)+δ(R(j),R(k))5:P(k)←{k,P(j)}6:**if**$t=y_{f}\left(k\right)$ of rectangle R(k)7:j← rectangle in L, with largest $y_{f}\left(j\right)\leq y_{f}\left(k\right)$8:**if** V(k)>V(j)9:**Insert** R(j) into L10:**Remove** all R(l) with V(l)≤V(k) and $y_{f}\left(l\right)\geq y_{f}\left(k\right)$11:$P^{*}=P\left(n^{*}\right)$ where $n^{*}=\arg\max_{n\in [1,N]}V\left(n\right)$12:**return**$P^{*}$

**Figure 3:**
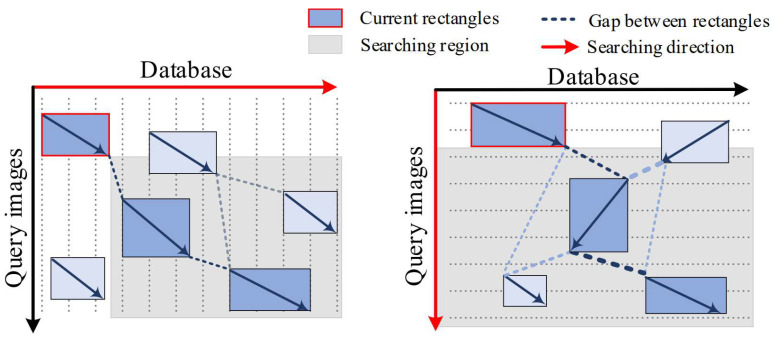
Comparison of existing and proposed rectangle chaining algorithms. The existing sparse DP algorithm (**left**) and the proposed algorithm (**right**). The proposed algorithm not only detects the robot’s forward motion but also detects backwardness using a rectangle chaining algorithm.

The proposed algorithm has the following improvements compared with the conventional method. First, the searching region for the next rectangle is expanded to deal with both the forward and backward moving of the robot as considered in recent papers [40,41]. Second, the searching direction is changed from the x-axis to y-axis, as the query images comes in time series and the next rectangle should be strictly below the current rectangle. Finally, the gap penalty is added to consider motion constraint of the robot. The final indices of rectangles L obtained through this algorithm is a set of *key fragments* that form the global path. Therefore, we can remove other local fragments that can cause a catastrophic failure when finding the global path as shown in Figure 3.

### 3.4. Global Sequence Alignment Using Anchors

Finally, the global path should be found by connecting key fragments in L found in the previous process. The starting and ending point of the key fragments are named *anchors* since they provide reliable clues to find the global path.

Let the set of key fragments is $L=\{R\left(p_{1}\right),R\left(p_{2}\right),\cdots ,R\left(p_{L}\right)\}$. Then, (L−1) paths starting from $\left(x_{f}\left(p_{k}\right),y_{f}\left(p_{k}\right)\right)$ to $\left(x_{i}\left(p_{k+1}\right),y_{f}\left(p_{k+1}\right)\right)$ should be found, where k=1,2,⋯,L−1. It is a kind of global alignment problem because the starting point and the ending point are fixed, and the task is to find a path connecting them. Any existing global alignment method can be used to fill the gap between key fragments in L. Note that we can find more accurate correspondences in the gap because the anchors provide the information of the starting and ending points of the sequences. In this paper, the DP-based method [33], which could consider various robot moving directions, is conducted to find the path between anchors.

Finally, the final global path is determined by the union of paths within the rectangles and paths connecting the anchors. Let the set of paths within rectangles is $\left\{A_{1},A_{2},\cdots ,A_{L}\right\}$ and the set of paths connecting the anchors is $\left\{B_{1},B_{2},\cdots ,B_{L-1}\right\}$. Then, the final global path is determined by alternatively concatenating the local sequences in key fragments and aligned sequences connecting the anchors as $\left\{A_{1},B_{1},\cdots ,A_{L-1},B_{L-1},A_{L}\right\}$. The proposed method has the advantage in overcoming false matches on featureless structures or partial failures during the alignment because it detects the paths with high reliability first and then connects them to find the final path. Therefore, it can perform accurate robot localization in changing environments.

## 4. Experimental Evaluation

### 4.1. Experimental Setup

**Figure 4:**
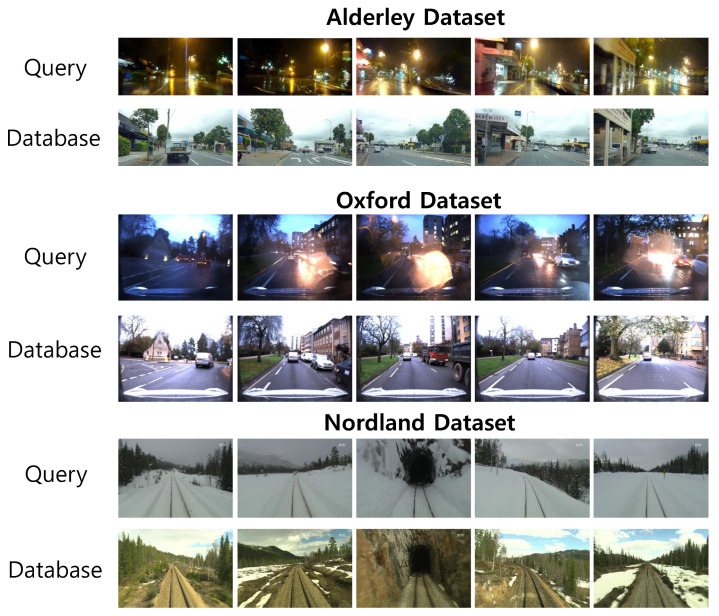
Sample image sequence taken from each of the 3 datasets: Alderley (day-to-night), Oxford (sunny-to-rainy), and Nordland (spring-to-winter).

To demonstrate the effectiveness of the proposed approach, three datasets were used from diverse environments. Since the proposed method aims to verify the performance of place recognition in changing conditions, the viewpoint changing problem is not considered. The Alderley dataset [15] consists of data collected during the days and nights on the same route. The daytime traverse was used as the database images and one nighttime traverse was used as the query image. The Oxford RobotCar dataset [42] consists of images taken on a sunny day and a rainy night in the city. The central images from the traverse ids 2014-11-25-09-18-32 and 2014-11-21-16-07-03 were used as database and query images, respectively. The final dataset is the Nordland dataset [43] collected from four seasons of rail journey. The spring–winter pair was used for our experiment. All images were resized to 224 × 224 and aligned to the same locations. In each dataset, 6000 images were used as a training set and 500 images were used as a test set. The sample image sequence of the datasets are shown in Figure 4. All experiments were implemented in Python 3.6.9 using the libraries Tensorflow 2.5.0 and carried out on a PC with a 3.9 GHz Intel Core-i7 CPU and 16 GB of main memory.

Our contributions are to propose a robust feature extraction based on VAE and a global alignment algorithm using the extracted features, so we divided the experiment into two main parts, the precision–recall performance of the features and the global alignment performances.

### 4.2. The Precision–Recall Performance of the Features

In the first part, the place recognition performance of the proposed feature was verified through precision–recall analysis. The relationship between precision and recall can be represented by a precision–recall curve, and the area under the curve (AUC) is a widely used metric for evaluating the place recognition performances. Precision is defined as the percentage of the number of correct matches for the number of total matches detected, and recall is the ratio of the number of correct matches to the total number of matches, that is:

(8) $$Precision=\frac{TruePositives}{TruePositives+FalsePositives}$$

(9) $$Recall=\frac{TruePositives}{TruePositives+FalseNegatives}$$

Our feature was compared with sum-of-absolute differences (SAD) from SeqSLAM [15], AlexNet [29], and NetVLAD [32]. The compared features demonstrated robust performance in changing environments, and widely used features in place recognition. Our proposed features were extracted from VAE model as shown in Table 1. Since the place recognition performance depends on the number of layers and nodes in the network, the best parameters were chosen through experiments.

**Figure 5:**
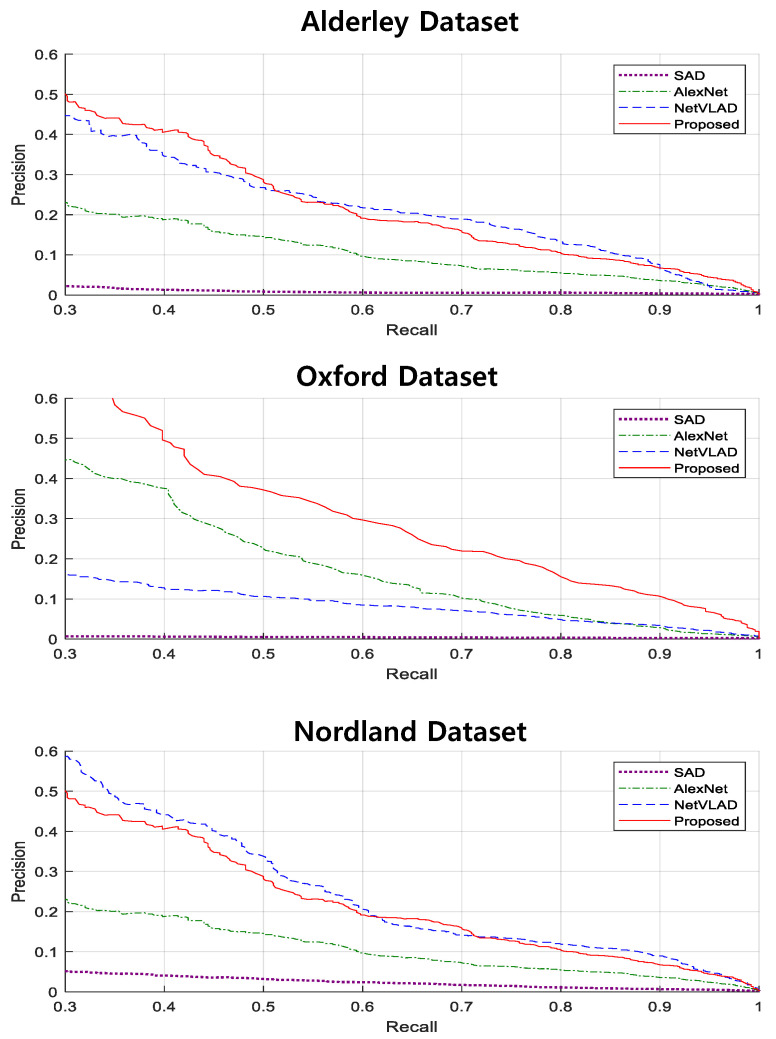
Sample image sequence taken from each of the three datasets: Alderley (day-to-night), Oxford (sunny-to-rainy), and Nordland (spring-to-winter).

The precision–recall results for each dataset are shown in Figure 5. In our test set, there is little difference in distance between frames, and as the environment changes extremely, most of the features do not exhibit high performance. However, our proposed feature showed comparable performance even when compared to the state-of-the-art feature, NetVLAD, and showed superior performance in the Oxford dataset. Since all feature extraction algorithms used the pretrained network, there was no significant difference in processing time.

The reason why our proposed feature performed so well on the Oxford dataset is that the images in this dataset were gray images, so the dimension was low. Our feature’s dimension is 128, as shown in Table 2, which is a very small number to contain the information of the whole image. However, compared to other features, it can be seen that our feature can efficiently store a lot of information despite its low dimensionality. Therefore, increasing the number of layers or nodes in the VAE can achieve high performance even with images of large dimensions.

In this experiment, the deep features from VAE were used, but there were limitations. Therefore, it is necessary to improve performance in combination with sequence-based global alignment method using the robot’s movement information.

### 4.3. Global Alignment Performance

**Figure 6:**
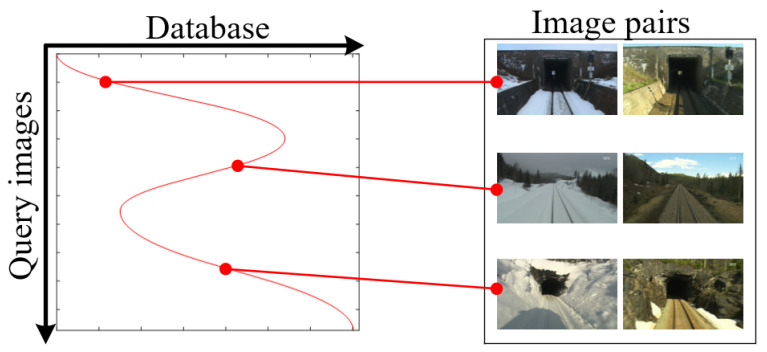
Ground truth of the corresponding frames and examples of matched frames.

To evaluate the global alignment performance, experiments were conducted on the Nordland dataset. We used the spring and winter sequences, and they were rearranged to generate various situations such as acceleration, deceleration, reverse moving, etc. The ground truth of the corresponding frames and examples of matched frames were shown in Figure 6.

**Figure 7:**
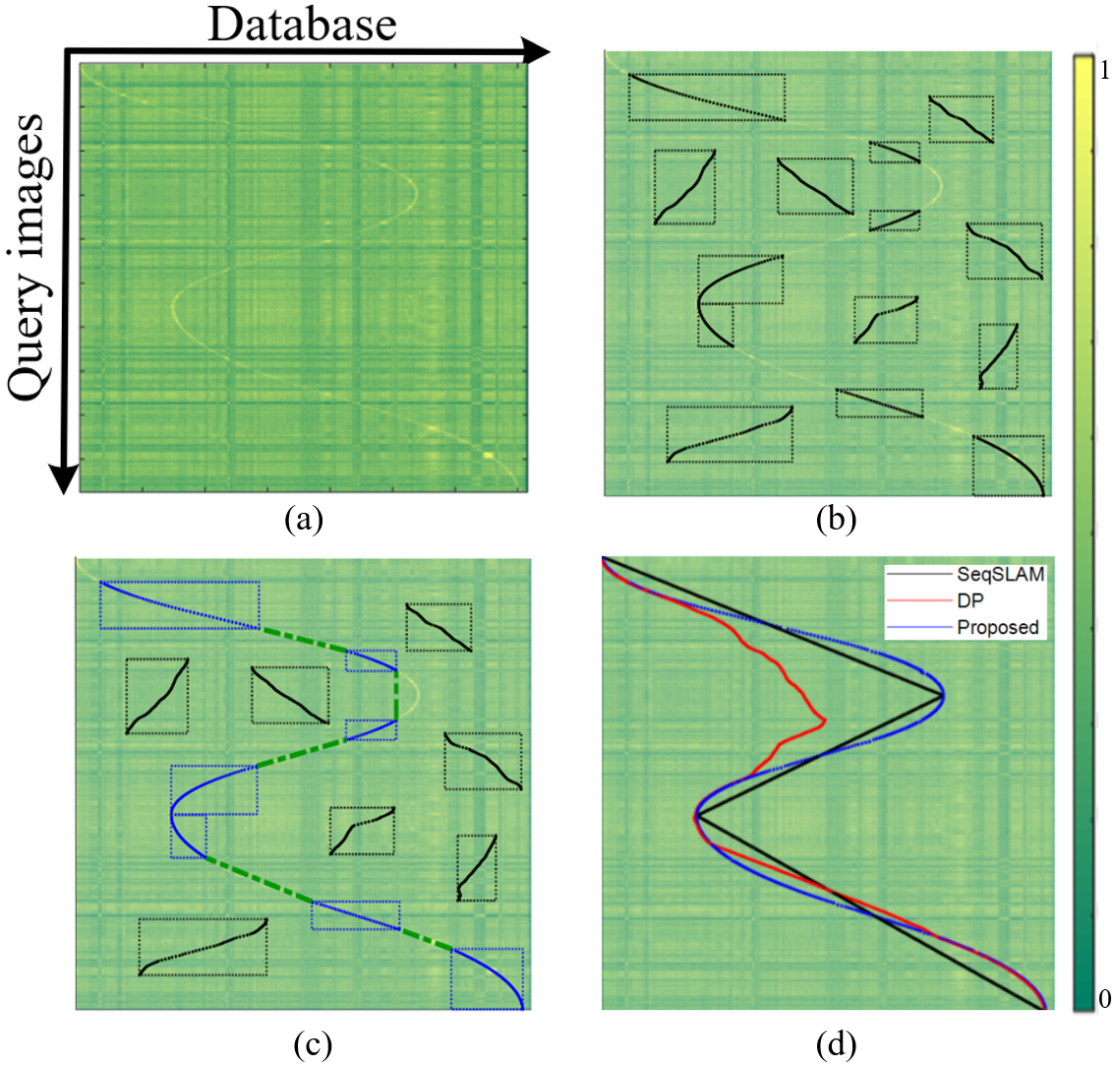
Experimental results: (**a**) constructed similarity matrix; (**b**) detected local fragments; (**c**) rectangle chained results; (**d**) global alignment results compared to other algorithms.

Experimental results are shown in Figure 7. First, local seeds were detected above the similarity 0.99 and performed local sequence alignment using the DP [33]. The local fragments above the weight scores 50 are chosen to be the candidates for the global alignment as shown in Figure 7b. Then, they are modeled as rectangles and connected using the proposed rectangle chaining algorithm. In Figure 7c, key sequences are represented as blue lines and the connections are shown as green dotted lines. Other local fragments represented as black lines are unnecessary. Finally, the global path estimated using the key sequences as anchors is shown in Figure 7d. To compare the performances of the proposed method, the resulting path of the SeqSLAM [15] and DP [33] are also presented.

We can conclude that the proposed method outperforms other algorithms, as SeqSLAM showed an inaccurate path due to the assumption of constant speed, and the DP partially failed to estimate the path in repetitive structures such as tunnels and roads. The precision–recall results and F1-scores in Table 2 also showed that the proposed method is more accurate than other methods.

## 5. Conclusions

To achieve robot localization in changing environments, the robust feature extraction method using the variational autoencoder was described to calculate the similarities between images. Then, the global sequence alignment method based on sparse DP was proposed to chain the reliable local fragments under the motion constraint of the robot. Experiments were performed on three datasets to demonstrate the effectiveness of the proposed approach. First, a precision–recall analysis was performed to test the robustness of the deep features, and the experimental results showed that the proposed features showed stable performance in various environments. In the Oxford dataset, the F1-score—which is the harmonic mean of the precision and recall—achieved 30% higher than that of AlexNet. In other datasets, the proposed feature achieved precision–recall results comparable to NetVLAD. Second, the global alignment performances were tested on the rearranged Nordland dataset. The false matches during the alignment were recovered, and the path of the robot was successfully estimated by using the proposed method. The precision–recall results showed that our method achieved more than three times higher performance than other methods.

Although the proposed method showed improved place recognition performance in appearance changing environments, another challenging environment in robot localization is the *viewpoint changing environment*. The viewpoint of the same place can change drastically when revisiting it, and finding correspondences between database and query images in this situation is challenging. Since the proposed autoencoder feature is a kind of global descriptors, it has limitations in dealing with viewpoint change problems compared to other local descriptor-based methods. In the future, it is necessary to improve our method to overcome the appearance changing problem as well as the viewpoint changing problem for practical robot localization.

## Figures and Tables

**Table 1:** The output shape of each layer in our VAE model.

Layer	Size	Layer	Size	Layer	Size	Layer	Size
conv1	112 × 112 × 32	conv4	14 × 14 × 128	fc7	2048	z_mean	128
conv2	56× 56 × 64	conv5	7 × 7 × 128	fc8	1024	z_var	128
conv3	28 × 28 × 64	fc6	4096	fc9	512	sampling	128

**Table 2:** The precision–recall and F1-score results.

Method	SeqSLAM [15]	DP [33]	Proposed
Precision	0.010	0.296	0.955
Recall	0.012	0.288	0.957
F1-score	0.011	0.292	0.956

**Figure A1:**
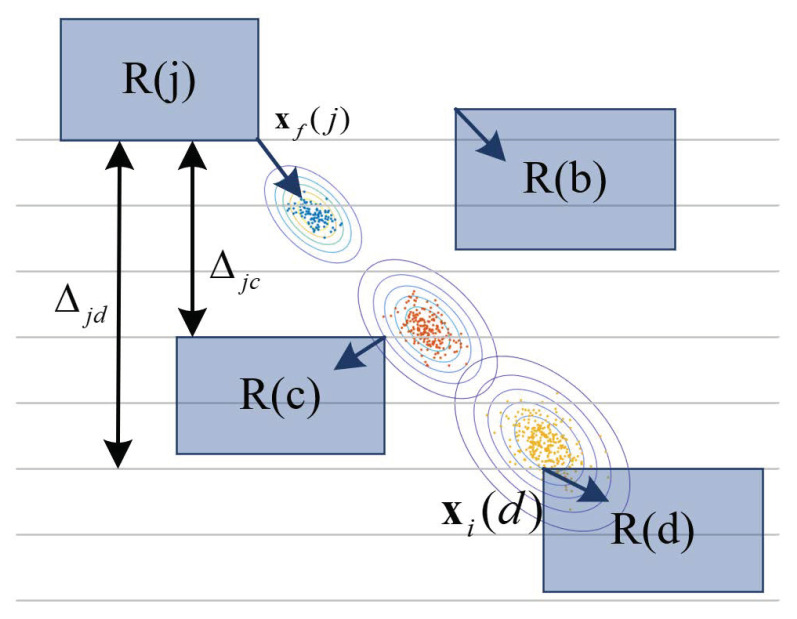
Gap penalty calculation using the linear robot motion model.

## Data Availability

Not applicable.

## Abbreviations

The following abbreviations are used in this manuscript:

SLAM	simultaneous localization and mapping
VAE	variational auto encoder
CAE	convolutional auto encoder
VLAD	vector of locally aggregated descriptors
CNN	convolutional neural network
RNN	recurrent neural network
DP	dynamic programming
SAD	sum of absolute differences

## Appendix A. Derivation of the Gap Penalty

The robot motion model assumes the true state at step *n* is evolved from the step at n−1 according to the following equation:

(A1) $$x_{n}=\;Fx_{n-1}+Bu_{n}+w$$

where F is the state transition matrix, B is the control model, w is the process noise which is assumed to be drawn from a multivariate normal distribution w∼N(0,Q).

In the model, the state vector is defined to have a position and velocity information as $x_{n}=\;\left[p_{n},v_{n}\right]$. Then, the linear motion model satisfies the following equation:

(A2) $$\left[\begin{array}{c} p_{n}\\ v_{n} \end{array}\right]=\;\left[\begin{array}{cc} I & \;tI\\ 0 & \;I \end{array}\right]\left[\begin{array}{c} p_{n-1}\\ v_{n-1} \end{array}\right]+w$$

where *t* is the sampling time. Then, *n*-steps after predicted states and covariances are as follows:

(A3) $$\begin{array}{cc} \mu_{n} & =\;F\mu_{n-1}\\ \; & =\;F^{n}\mu_{0}\\ \Sigma_{n} & =\;F\Sigma_{n-1}F^{⊤}+Q\\ \; & =\;F^{n}\Sigma_{0}{\left(F^{⊤}\right)}^{n}+\sum_{k=0}^{n-1}F^{k}Q{\left(F^{⊤}\right)}^{k}. \end{array}$$

As the predicted state after *n* steps follows $x_{n}∼N\left(\mu_{n},\Sigma_{n}\right)$, the probability of the current state when given initial state and the number of steps after initial state is calculated.

Our purpose is to calculate the gap penalty between rectangles as shown in Figure A1. From the view of the R(a), the purpose is to calculate the gap penalty between R(b), R(c), and R(d). As R(a) is the initial state, the expected position of the robot can be seen by the evolution of the Gaussian distribution.

The initial state is at the ending point of R(j), which is $x_{0}={\left[p_{f}\left(j\right),v_{f}\left(j\right)\right]}^{T}$ and the final state is at the starting point of the candidate rectangle R(k), $x={\left[p_{i}\left(k\right),v_{i}\left(k\right)\right]}^{T}$. Therefore, the step size is $\Delta =|y_{f}\left(j\right)-y_{i}\left(k\right)|$, the gap penalty becomes the following equation:

(A4) $$\delta \left(R\left(j\right),R\left(k\right)\right)=\;C|\Sigma_{\Delta }|\exp{\left(-\frac{1}{2}\left({\left(x-\mu_{\Delta }\right)}^{T}\Sigma^{-1}\left(x-\mu_{\Delta }\right)\right)\right)}^{-1}$$

where x is the state at R(k) and *C* is the weight factor.

Let us consider the Figure A1 case. R(b) does not need to be considered since $y_{f}\left(j\right)<y_{i}\left(d\right)$. In the case of R(c), the Gaussian distribution corresponding to the $\Delta_{jc}$ is represented as red dots. However, the state $x_{c}={\left[p_{i}\left(c\right),v_{i}\left(c\right)\right]}^{T}$ is far from the center of the Gaussian distribution, and it can be predicted that it will have a low probability. On the other hand, the Gaussian distribution corresponding to the $\Delta_{jd}$ is represented as yellow dots in the case of R(d), and it can be found that the state $x_{d}={\left[p_{i}\left(d\right),v_{i}\left(d\right)\right]}^{T}$ is near the center of the distribution. The penalty is defined as the inverse of the probability, and it is concluded that δ(R(j),R(c))>δ(R(j),R(d)).
